# Integrated epigenomic analysis stratifies chromatin remodellers into distinct functional groups

**DOI:** 10.1186/s13072-019-0258-9

**Published:** 2019-02-12

**Authors:** Katherine A. Giles, Cathryn M. Gould, Qian Du, Ksenia Skvortsova, Jenny Z. Song, Madhavi P. Maddugoda, Joanna Achinger-Kawecka, Clare Stirzaker, Susan J. Clark, Phillippa C. Taberlay

**Affiliations:** 1 Epigenetics Research, Genomics and Epigenetics Division, Garvan Institute of Medical Research, Sydney, NSW 2010 Australia; 2 St Vincent’s Clinical School, UNSW Australia, Sydney, NSW 2000 Australia; 3 School of Medicine, Collage of Health and Medicine, University of Tasmania, Hobart, TAS 7000 Australia

**Keywords:** Chromatin, Nucleosome, Chromatin remodelling, Enhancer, Promoter, Gene regulation, Epigenetics, CHD, SWI/SNF, INO80, ISWI

## Abstract

**Background:**

ATP-dependent chromatin remodelling complexes are responsible for establishing and maintaining the positions of nucleosomes. Chromatin remodellers are targeted to chromatin by transcription factors and non-coding RNA to remodel the chromatin into functional states. However, the influence of chromatin remodelling on shaping the functional epigenome is not well understood. Moreover, chromatin remodellers have not been extensively explored as a collective group across two-dimensional and three-dimensional epigenomic layers.

**Results:**

Here, we have integrated the genome-wide binding profiles of eight chromatin remodellers together with DNA methylation, nucleosome positioning, histone modification and Hi-C chromosomal contacts to reveal that chromatin remodellers can be stratified into two functional groups. Group 1 (BRG1, SNF2H, CHD3 and CHD4) has a clear preference for binding at ‘actively marked’ chromatin and Group 2 (BRM, INO80, SNF2L and CHD1) for ‘repressively marked’ chromatin. We find that histone modifications and chromatin architectural features, but not DNA methylation, stratify the remodellers into these functional groups.

**Conclusions:**

Our findings suggest that chromatin remodelling events are synchronous and that chromatin remodellers themselves should be considered simultaneously and not as individual entities in isolation or necessarily by structural similarity, as they are traditionally classified. Their coordinated function should be considered by preference for chromatin features in order to gain a more accurate and comprehensive picture of chromatin regulation.

**Electronic supplementary material:**

The online version of this article (10.1186/s13072-019-0258-9) contains supplementary material, which is available to authorized users.

## Background

Chromatin is a dynamic and multi-layered structure of which the core building block is the nucleosome. Nucleosomes are comprised of an octamer of histone proteins and 147 base pairs (bp) of DNA in approximately two helical turns [1]. The unique chromatin conformation of any given cell is typically maintained throughout divisions and serves as a physical barrier to transcription factors and other regulatory proteins in order to prevent promiscuous gene expression [2, 3]. Thus, chromatin structure must be modulated for regulatory factors to access DNA when required. This is largely achieved through the movement of nucleosomes by ATP-dependent chromatin remodelling complexes, which utilise ATP hydrolysis to organise nucleosomes into an active (relaxed) or repressive (compact) conformation. The process of chromatin remodelling provides means for regulating DNA structure with precision and accuracy to facilitate diverse cellular processes including transcriptional regulation, DNA repair, DNA replication and cell cycle progression [2, 4]. However, despite growing research into the molecular and biochemical mechanisms of chromatin remodelling, it is still not completely understood how remodelling complexes work together to position nucleosomes for the required chromatin function.

Beyond the physical nature of its structure, chromatin carries diverse gene regulatory information including post-translational modifications of histone proteins and DNA methylation [2, 4, 5]. These features, together with non-coding RNA (ncRNA) species, form the epigenome. Chromatin remodellers are drawn to their target regions by sequence-specific regulatory proteins or ncRNAs [6–8] and use their protein structural domains to identify epigenetic patterning and the ‘linker’ DNA between nucleosomes to identify their preferred nucleosome substrate [3, 9–11]. Yet, it is important to consider that many regulatory regions of the genome are a composite of multiple epigenetic marks; therefore, there is a multifaceted relationship between chromatin remodellers and the epigenome. For example, bivalent promoters are characterised by the trimethylation of histone 3 at both lysine 4 (H3K4me3) and lysine 27 (H3K27me3) [12–14] and could therefore potentially be ‘read’ by remodellers recognising either of these marks. Additionally, these relationships are not linear as more than one remodeller may recognise a single histone modification [15]. Uncovering the extent of overlapping and unique activity of chromatin remodeller proteins is of great interest and is essential for understanding the influence of the epigenetic signature on chromatin remodelling events at any given locus.

**Fig. 1:**
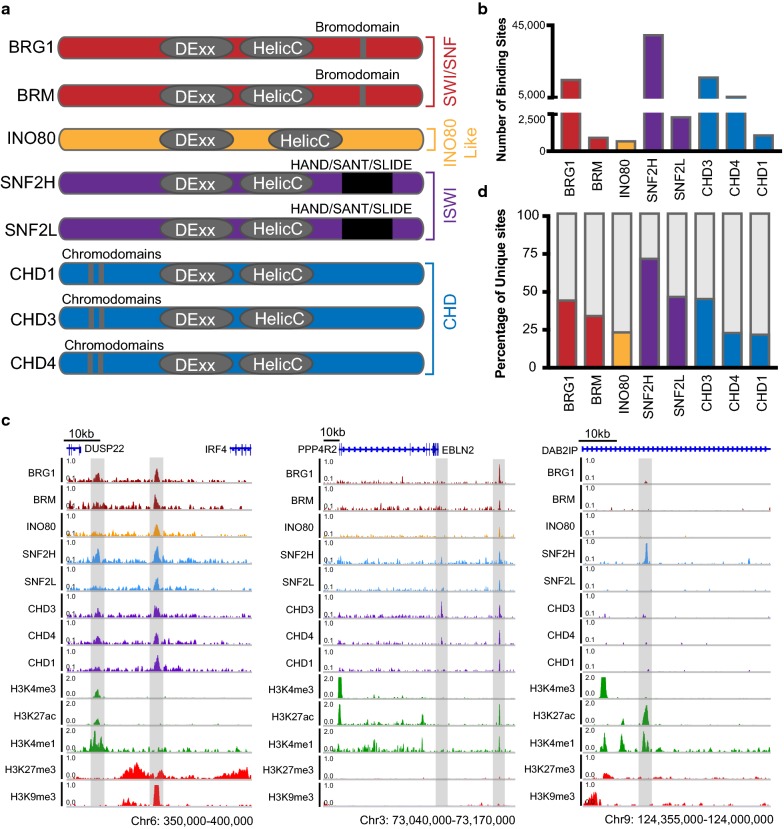
Chromatin remodeller occupancy and expression in LNCaP cells. **a** The domain organisation of each ATPase catalytic subunits of chromatin remodelling complexes used in this study. Each contains a SNF2-like helicase comprising of DEXDc and HELICc domains, with each subfamily containing additional domains. Key domains defining each subfamily are shown. **b** Number of binding sites occupied by each remodeller from MACS2 peak calling. **c** Genome browser view of chromatin remodeller occupancy and key histone modification tracks in LNCaP cells with overlap of chromatin remodeller binding and where SNF2H is the only bound remodeller. **d** Percentage of remodeller protein-binding sites unique to each remodeller (coloured) and percentage that overlaps with one or more of the other remodeller proteins (grey)

There are four structural families of chromatin remodellers: switch/sucrose non-fermenting (SWI/SNF), imitation switch (ISWI), inositol requiring 80-like (INO80-like) and chromodomain helicase DNA binding (CHD) [3, 16–18]. Chromatin remodelling complexes share common features such as an essential catalytic ATPase and a high affinity for nucleosomes [2, 3, 16–18], yet the ATPases within these complexes have evolved unique features that pertain to their specific function. SWI/SNF ATPases contain bromodomains for recognising acetylated histones; ISWI ATPases contain a HAND/SANT/SLIDE domain for recognising internucleosomal DNA, the INO80-like ATPases have a longer peptide chain between their two helicase domains that has been proposed to fit Holliday junctions and replication forks, and the CHD ATPases contain chromodomains [16, 18–22] (Fig. 1a). It is known that remodellers can have both cooperative and opposing roles at the same genomic location in yeast and mice [23–27]; however, the extent of this has not been studied extensively in human cells. Importantly, there has been no study to date examining more than three remodellers concurrently in human cells, nor with simultaneous consideration of the epigenome, transcriptome and chromatin structural states. Moreover, the influence of the epigenome on remodeller function is not well understood.

Here, we have examined the binding profiles of eight different chromatin remodeller proteins and integrated these with extensive epigenomic data, including histone modifications, DNA methylation and chromosome architectural information. Our study reveals that chromatin remodellers can be stratified into two groups based on their binding enrichment at either ‘actively marked’ or ‘repressively marked’ regions and their interactions with chromatin features of the epigenome.

## Results

### The degree of chromatin remodeller binding correlates with the remodeller gene expression levels in prostate cancer cells

To improve our understanding of the relationship between different chromatin remodellers, we sought to examine the genomic binding profiles of multiple chromatin remodeller proteins simultaneously from publicly available data obtained from LNCaP prostate cancer cells [28]. All eight remodellers examined are catalytic ATPases that form mutually exclusive complexes and together represent all four the structural families, unlike previous studies in mouse embryonic stem cells. The remodellers studied were BRG1 and BRM from the SWI/SNF family; SNF2H and SNF2L from the ISWI family; INO80 from the INO80-like family; and CHD1, CHD3 and CHD4 from the CHD family (Fig. 1a). We first assessed the gene expression level of each remodeller using published RNA-seq data from LNCaP cells [29] and compared this to prostate tumour samples from The Cancer Genome Atlas (TCGA) database [30] to determine the validity of using LNCaP cells as a prostate cancer model to explore chromatin remodellers. We found a high concordance in the gene expression pattern for each remodeller between these data sets, where those genes displaying higher expression in clinical samples from TCGA (*n* = 486) also had higher expression in the LNCaP cell line (Additional file 1: Figure S1A–B). A similar comparison was made between a normal prostate epithelial cell line (PrEC) and the normal samples from TCGA (*n* = 52); the expression patterns of the remodellers in PrEC cells mirrored that of the normal clinical samples (Additional file 1: Figure S1C–D). We calculated the Pearson correlation coefficient and found a strong positive linear relationship between the LNCaP cells and the TCGA tumours (*Pearson R *=0.8375552) and between the PrEC cells and the TCGA normal data sets (*Pearson R *=0.670013; Additional file 1: Figure S1E). The exception to this was SNF2L, which was significantly lower in LNCaP cells compared to the TCGA tumours.

Using the ChIP-seq binding profiles of the eight chromatin remodellers from Ye et al. [28], we next examined the number of individual binding sites for each remodeller and established they ranged from 712 for INO80 to 39,887 for SNF2H (Fig. 1b), and in total, there were 60,043 genomic regions bound by at least one chromatin remodeller. Upon visualising the remodeller binding sites, we observed several regions where multiple remodellers were bound, such as the intergenic region between *DUSP22* and *IRF4* on chromosome 6 and upstream of the *EBLN2* gene on chromosome 3 (Fig. 1c). Additionally, there were several sites where a single remodeller was bound, such as CHD3 at the *EBLN2* gene promoter and SNF2H within the *DAB2IP* gene (Fig. 1c). We observed that the majority of the binding sites occurred over regions marked by gene regulatory histone modifications. Over 75% of the remodeller binding sites were under 750 bp in size, corresponding to a span of ~ 1–5 nucleosomes (Additional file 1: Figure S2A–H), covering 0.93% of the genome. The degree of overlap between the remodellers varied extensively with the number of unique binding sites for each remodeller ranging from ~ 25 to ~ 50%, with the exception of SNF2H which had ~ 75% unique sites, likely paralleling the overall large number of binding sites for this remodeller (Fig. 1d).

Together, these data show that there is large variation in the number of genomic sites occupied by chromatin remodellers, with both independent and a high degree of overlapping activity between the remodeller proteins. Importantly, the high overlap in the number of binding sites containing at least two remodellers suggests there is widespread coordinated activity across the genome.

### The epigenome stratifies chromatin remodellers into two groups: Group 1 is associated with ‘actively marked’ chromatin and Group 2 with ‘repressively marked’ chromatin

**Fig. 2:**
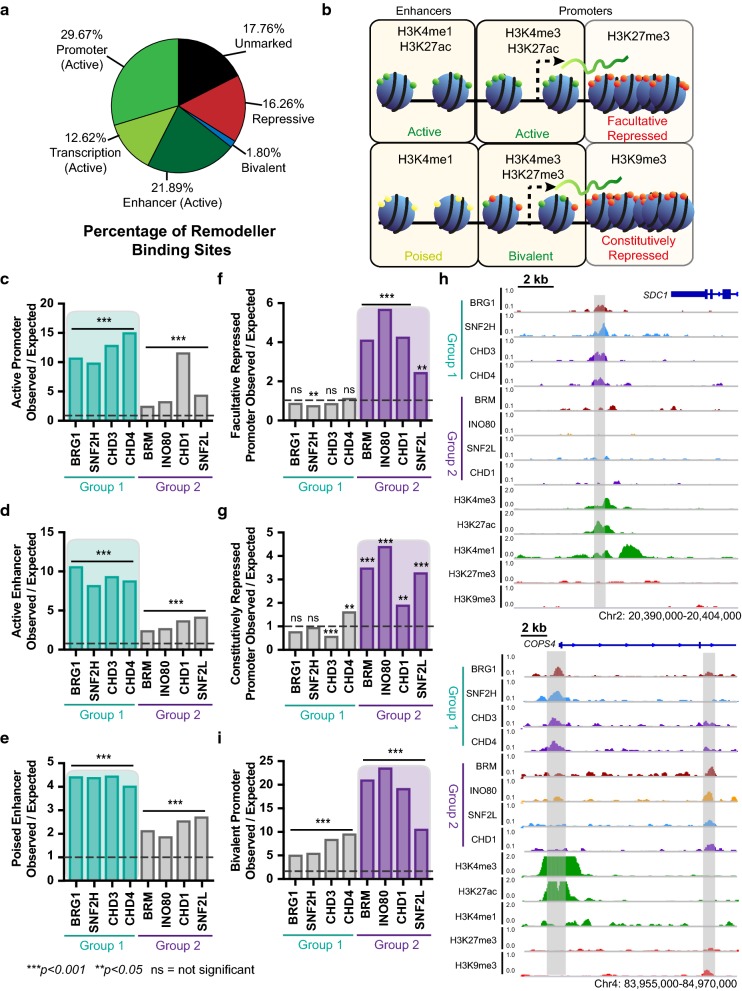
Chromatin remodellers bind to distinct regions of chromatin in two defined groups. **a** Percentage of all 60,043 remodeller binding sites in each chromHMM chromatin state compiled into active promoters (promoters and flanking promoters), transcription (strong and weak and at 5′/3′ ends of genes), active enhancers (genic and other enhancers), bivalent (enhancers, promoters, and flanking promoters), repressive (polycomb, weak polycomb, zinc finger repeats and heterochromatin), and unmarked (no core marks from the Roadmap Epigenome chromatin state model). **b** Schematic diagram of histone modifications present at ‘active’ and ‘repressive’ promoters and enhancers. ‘Active’ histone modifications are shown in green with increased spacing between nucleosomes, while ‘repressive’ histone modifications are shown in red with compacted nucleosomes. **c**–**g**, **i** Enrichment of chromatin remodeller binding sites at key gene regulatory features defined by ‘active’ and ‘repressive’ histone modifications. Significant enrichment is defined as a score above one and significantly depleted as below one with Benjamini–Hochberg adj *p* value, ***p < 0.001 or **p < 0.05. **h** Genome browser view of chromatin remodeller occupancy and key histone modification tracks in LNCaP cells showing upstream of the *SDC1* promoter and at the *COPS4* promoter, which is occupied by all Group 1 remodellers and adjacent to exon 2 in COPS4 that is occupied by all Group 2 remodellers

Chromatin remodellers do not exhibit sequence specificity in their binding and therefore have the potential to bind anywhere across the genome. However, as the majority of remodeller proteins contain domains targeting nucleosomes with post-translational histone tail modifications [2, 31–33], it is expected that the bulk of binding will occur within histone ‘marked’ chromatin. We compiled histone modification ChIP-seq data sets from LNCaP cells [29, 34] and performed a chromatin multivariate hidden Markov model (chromHMM) analysis to determine chromatin states based on the Epigenome Roadmap core model for chromatin state discovery [35] (Additional file 1: Figure S3A-B; see “Methods”). This analysis found that of the histone modifications examined, the ‘marked’ chromatin (active, bivalent and repressive states) encompassed 50.1% of the genome, while 49.9% is not marked with any of the histone modifications included in the analysis (see “Methods”). Of the 60,043 remodeller binding sites, 64.18% fell within ‘actively marked’ chromatin (promoters, enhancers and transcriptionally active), 16.26% fell within ‘repressively marked’ chromatin and 1.80% within bivalent chromatin. Together, 82.24% of all remodeller binding occurred within ‘marked’ chromatin, confirming that remodellers are largely localised to regulatory regions of the genome and suggesting that the primary role of remodellers is to establish and maintain gene expression (Fig. 2a).

As our data show that the majority of chromatin remodeller binding occurs at histone ‘marked’ chromatin, we next examined the enrichment of remodeller binding across gene regulatory features. We specifically targeted ‘actively marked’ and ‘repressively marked’ regulatory elements that are ‘marked’ with a composite of histone modifications. To define these regulatory chromatin features, we used annotated transcription start sites (TSS) from GENCODE and classified the promoter as a 2 kb region surrounding the TSS. We then separated these promoters according to the histone modifications present in LNCaP cells to classify them as active (H3K4me3 and H3K27ac), facultative repressed (H3K27me3), constitutively repressed (H3K9me3) and bivalent (H3K4me3 and H3K27me3) (Fig. 2b). Additionally, we defined putative enhancers as being at least 2 kb away from an annotated TSS and classified them as either active (H3K4me1, H3K27ac, p300 and DNaseI accessible; Additional file 1: Figure S3C) or poised (H3K4me1) (Fig. 2b), and analysed the chromatin remodellers at these regions.

We assessed whether each chromatin remodeller was bound at the gene regulatory features defined above, more (enriched) or less (depleted) than expected by chance using the Genome Association Tester (GAT) [36]. All chromatin remodeller proteins were significantly enriched at ‘actively marked’ regulatory elements, (Fig. 2c–e; Additional file 1: Figure S4A). However, it was notable that they could be stratified into two distinct groups based on the level of enrichment at these regions. BRG1, SNF2H, CHD3 and CHD4, herein called Group 1, were significantly more enriched compared to BRM, INO80, SNF2L and CHD1, herein called Group 2 (Student’s unpaired *T* test, active promoters *p* = 0.04; active enhancers *p *= 0.0001; poised enhancers *p* = 0.0004). At gene regulatory regions marked by ‘repressively marked’ epigenetic features, the facultative and constitutively repressed promoters, the majority of Group 1 remodellers were not significantly enriched, in contrast to Group 2 (Fig. 2f, g). Concomitantly, there was a significant difference between the mean enrichment scores of Group 1 and Group 2 remodellers at repressed regions (Student’s unpaired T-test, facultative repressed promoters *p* = 0.016; constitutively repressed promoters *p* = 0.02). However, statistical enrichment at a particular genomic annotated feature does not determine the extent of direct overlap of remodeller binding sites at these features. By examining the direct overlap within each group of remodellers, we found that there are common and unique binding sites (Additional file 1: Figure S4B-C); for Group 1 binding sites, only 22.6% contained two or more Group 1 remodellers (Additional file 1: Figure S4D), and for Group 2 binding sites, only 17.3% contained more than one Group 2 remodellers (Additional file 1: Figure S4E). Example regions bound by all Group 1 remodellers are illustrated upstream of the *SDC1* promoter and at the *COPS4* promoter, and adjacent to exon 2 of the *COPS4* gene for Group 2 (Fig. 2h). Therefore, while each group of remodellers was enriched at the same genomic features (i.e. binding occurs higher than expected by chance), there are only a small percentage of regions where all the remodellers bind together, highlighting varied roles for these remodellers.

We next examined bivalent chromatin, which exhibits both active and repressive epigenetic features. Given the above results, we hypothesised that both Group 1 and Group 2 remodellers would be enriched equally at bivalent chromatin. We indeed found that all remodellers were significantly enriched at bivalent promoters, and notably, we observed that Group 2 remodellers had significantly higher enrichment compared to Group 1 (Fig. 2i; Student’s unpaired T-test, *p *=0.0004). This is in line with previous research showing low enrichment of BRG1 and CHD4 at bivalent chromatin [27].

We next performed the equivalent enrichment analysis using the chromHMM 15 state model (see “Methods”). Again, Group 1 remodellers had a mean enrichment score significantly higher than Group 2 at ‘actively marked’ regions and a lower score for ‘repressively marked’ regions (Additional file 1: Figure S5A-I), with the exception of intragenic enhancers. All remodellers were enriched across all three bivalent states in the model (bivalent promoter, flanking bivalent promoter and bivalent enhancer), but there was no significant difference between the Group 1 and Group 2 (Additional file 1: Figure S5 J-L; Student’s unpaired T-test, *p *=0.118). The chromHMM model also defines states of active transcription, and we found that there is no significant enrichment of remodellers in the regions flanking active transcription and all remodellers were significantly depleted from regions of active transcription (Additional file 1: Figure S5M–O). Moreover, as there is also no significant enrichment at intragenic enhancers (Additional file 1: Figure S5D), we speculated that sites of active transcription (including the intragenic enhancers) may be due to the highly dynamic and transient nature of transcription, preventing a stable signal of the chromatin remodellers from being detected within these regions.

An interesting exception to the stratification of the remodellers described above is the presence of CHD1 at ‘actively marked’ promoters. At both annotated ‘actively marked’ promoters (Fig. 2c) as well as promoters defined by the chromHMM segmentation (Additional file 1: Figure S5A), CHD1 is found to be significantly enriched at a level comparable to the Group 1 remodellers. As CHD1 does not display the same level of significant enrichment at any other ‘actively marked’ regulatory regions, we speculate that non-epigenetic factors may be driving this high level of promoter binding.

We then took a second approach where we tested the average distribution of the ChIP-seq signal of key gene regulatory histone modifications: H3K4me3, H3K4me1, H3K27me3 and H3K9me3, across the remodeller binding sites (Additional file 1: Figure S6A–D). Our results demonstrated that the active histone marks, H3K4me3 and H3K4me1, displayed a higher average signal across the binding sites of Group 1 remodellers, and repressive histone marks exhibited a higher average signal across Group 2 remodellers. This further confirms the association of Group 1 with ‘actively marked’ regions and Group 2 with ‘repressively marked’ regions. Additionally, at ‘actively marked’ promoters and bivalent promoters defined by these histone modifications, the Pearson correlation coefficient between the remodellers in Group 1 was higher compared to Group 2 (Additional file 1: Figure S6E–F), indicating that there is more similarity in the binding pattern between Group 1 compared to Group 2 at active regions. Furthermore, we found the converse was true for repressed promoters (Additional file 1: Figure S6G–H). Taken together, these data suggest that while all remodellers are associated with ‘actively marked’ chromatin states, Group 1 remodellers have a more pronounced role at these ‘actively marked’ regions, while Group 2 remodellers play a greater role at ‘repressively marked’ regions.

### Chromatin remodellers bind to AT-rich DNA and are found at unmethylated regions

We were interested to know whether DNA methylation or the DNA sequence within the remodeller binding site could also stratify the chromatin remodellers into Group 1 and 2. Although chromatin remodellers are responsible for positioning nucleosomes, the genome-wide patterning of nucleosomes is also determined in part by the DNA sequence, where there is a preference for DNA rich in ApT and TpA dinucleotides that are able to bend more sharply around the histone octamer [1, 37, 38]. We calculated the density of all four nucleotides within each chromatin remodeller binding site and found a higher density of A and T nucleotides within the remodeller binding sites for all of the remodellers, and no difference between Group 1 and Group 2 (Additional file 1: Figure S7A-B). Additionally, the ApT and TpA dinucleotides within Group 1 and Group 2 remodeller binding sites occur as frequently as all other dinucleotides in the genome, except for CpG dinucleotides (Additional file 1: Figure S7C–D). Together, this suggests that while intrinsic nucleosome positioning is determined in part by sequence composition, chromatin remodeller nucleosome targeting is not dependent on overall DNA sequence composition, nor does the sequence stratify remodellers into Group 1 and Group 2.

DNA methylation of cytosine residues occurs primarily in a CpG context and has a complex role. At promoters, it is associated with chromatin compaction, but in gene bodies it is associated with active expression [39–44]. We next examined whether DNA methylation was present at the remodeller binding sites. Overall, we detected very low levels of DNA methylation across all remodeller binding sites (Additional file 1: Figure S7E). However, we found that remodellers bound to regions with a higher CpG density had less DNA methylation within their binding sites and conversely regions with lower overall CpG density displayed higher the levels of DNA methylation (Additional file 1: Figure S7E–F).

**Fig. 3:**
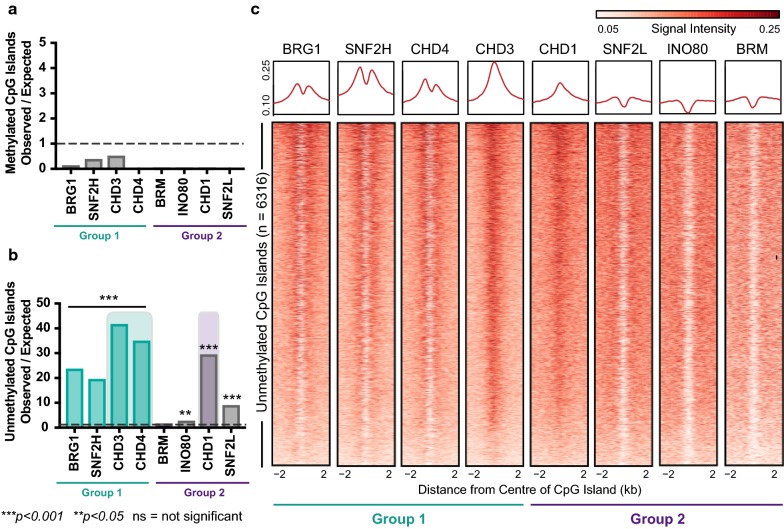
Chromatin remodellers do not stratify into Group 1 and Group 2 at CpG islands. **a**, **b** Enrichment of chromatin remodellers at methylated and unmethylated CpG islands. Significant enrichment is defined as a score above one and significantly depleted as below one with Benjamini–Hochberg adj *p* value, ***p < 0.001 or **p < 0.05. **c** Heatmap of remodeller ChIP-seq signal at unmethylated promoter CpG islands, ± 2 kb from the centre of the island, with average profile plots shown above the heatmap

CpG islands are defined as regions of DNA with high CpG density stretching beyond 500 bp [45]. A list of annotated CpG islands was obtained from UCSC, and promoter CpG islands defined as the intersection between CpG islands and Ensembl transcription start sites (see “Methods”). These were then divided into methylated (average methylation across the CpG island above 50%) or unmethylated using whole-genome bisulphite sequencing (WGBS) data generated from LNCaP cells [46]. There was a small, but significant difference in the CpG density of the methylated and unmethylated CpG islands (one-way ANOVA, *p *<0.001; Additional file 1: Figure S7G). By performing GAT statistical enrichment analysis, we found that methylated promoter CpG islands are depleted of all chromatin remodellers (Fig. 3a). This was due to only 12 total remodeller peaks (the sum of: one peak for BRG1, six peaks for CHD3 and five peaks for SNF2H) overlapping methylated CpG islands. All eight remodellers displayed varying levels of positive enrichment at unmethylated promoter CpG islands (Fig. 3b), with the CHD family (both CHD3 and CHD4 from Group 1 and CHD1 from Group 2) showing the highest level of enrichment. We next examined the ChIP-seq signal intensity of the remodellers across unmethylated promoter CpG islands, extending to ± 2 kb from the centre of the island. Interestingly, we noted that there was variation in the binding pattern of remodellers. CHD1 and CHD3 bound closer to the centre of the island, BRG1, CHD4 and SNF2H bound at the borders and BRM, INO80 and SNF2L were depleted in the centre (Fig. 3c). This suggested that chromatin remodellers are associated with maintaining chromatin organisation at different parts of promoter CpG islands, and we propose that they work in coordination to maintain the accessibility status of the island; however, the remodellers do not stratify into Group 1 and Group 2 based on whether the island is methylated or unmethylated.

### Chromatin preferentially occupied by Group 1 remodellers is nucleosome depleted

**Fig. 4:**
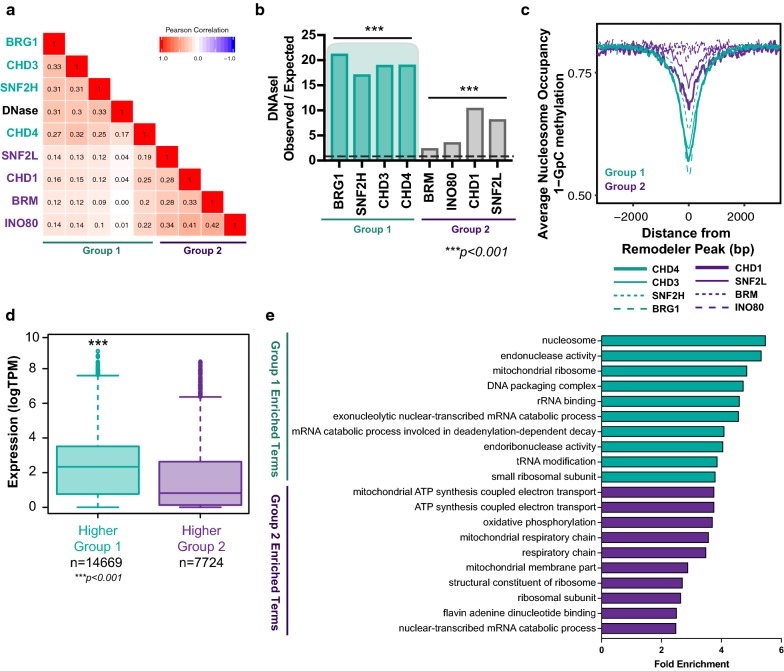
Group 1 chromatin remodellers highly overlap accessible DNA regions. **a** The ChIP-seq signal of the chromatin remodellers and the signal from the ENCODE DNAseI peaks were determined at DNAseI called sites, and then Pearson’s correlation scores were calculated between each data set and compiled into a matrix. The matrix was ordered by hierarchical clustering using the distance between correlation scores. This resulted in the Group 1 remodellers clustering with the DNAseI data and Group 2 remodellers clustering separately. Samples are presented in the same order on both the *x*- and *y*-axis. **b** Enrichment of chromatin remodeller binding sites at DNAse1 sites. Significant enrichment is defined as a score above one and significantly depleted as below one with Benjamini–Hochberg adj p-value, ***p < 0.001 or **p < 0.05. **c** Nucleosome occupancy defined from NOMe-seq ± 2 kb from the centre of chromatin remodeller binding sites. **d** Boxplot of the relationship between gene expression (TPM) and remodeller binding at promoters of genes that are expressed in LNCaP cells. Expressed genes were divided into genes that have a higher enrichment of Group 1 remodellers (green) at their promoter and those that have a higher enrichment of Group 2 remodellers (purple) at their promoter. The expression of Group 1 is significantly higher (*p* < 0.001, Wilcox rank sum, one-tailed) than Group 2. **e** Enrichment scores of the top 10 GO terms for promoters with a higher signal of Group 1 remodellers (Green) or a higher signal of Group 2 remodellers (purple)

Nucleosome occupancy at regulatory features naturally represses transcription by creating an inhibitory structure, and therefore, regions of ‘actively marked’ chromatin are typically depleted of nucleosomes and have increased rates of nucleosome turnover and greater spacing between nucleosomes. Since Group 1 remodellers are enriched at ‘actively marked’ chromatin regulatory regions, we next asked whether the genomic regions corresponding to Group 1 localisation are depleted of nucleosomes. First, we examined sites of nucleosome depletion from DNaseI hypersensitivity data from LNCaP cells [47]. Analysis of the correlation between chromatin remodeller presence and DNaseI sites revealed that Group 1 remodeller occupancy is highly correlated with DNaseI hypersensitive sites (Fig. 4a), in addition to DNaseI sites being highly enriched for Group 1 remodellers (Fig. 4b). Given that DNaseI digestion does not detect all sites of ‘open’ chromatin [48], we assessed the link between chromatin remodeller binding and accessible chromatin using an alternative method—nucleosome occupancy and methylation sequencing (NOMe-seq). NOMe-seq [48–50] uses exogenous methylation of GpC dinucleotides to identify accessible chromatin regions devoid of nucleosomes or tight binding transcription factors. Importantly, this method also avoids cleavage bias found in DNaseI data [51]. We found that GpC methylation was higher at Group 1 remodeller binding sites compared to Group 2, suggesting that genomic regions occupied by Group 1 remodellers contain fewer nucleosomes than those regions occupied by Group 2 remodellers (Fig. 4c). These data are consistent with the preference of Group 1 remodellers for ‘actively marked’ chromatin states and for Group 2 remodellers to localise to regions of more ‘repressively marked’ chromatin.

Accessible chromatin is associated with active regulatory elements and gene activity. Therefore, to determine whether chromatin remodeller binding was correlated with gene expression in LNCaP cells, the ChIP-seq read counts of all Group 1 and Group 2 remodellers were pooled. Then, the difference between the read counts (signal) for Group 1 remodellers compared to Group 2 remodellers at the promoters of expressed genes was calculated. Genes were separated into those with a higher level of Group 1 chromatin remodeller binding and those with a higher level of Group 2 remodeller binding at their promoters and the level of expression for each group plotted as transcripts per million reads (logTPM). Of the 22,393 genes that were actively expressed in LNCaP cells, 14,669 had a higher signal for Group 1 chromatin remodellers and 7724 had a higher signal for Group 2 chromatin remodellers at their promoters. Genes with a higher level of Group 1 remodellers had higher expression compared to those that had a higher expression of Group 2 remodellers (Fig. 4d), supporting the conclusion that Group 1 remodellers are associated with increased gene activity. We performed a gene ontology (GO) analysis of active genes that more highly bound by either Group 1 or Group 2 remodellers using GREAT [52]. For gene promoters with higher Group 1 binding, the most significant GO terms enriched were related to the nucleosome, transcriptional processes such as rRNA binding and tRNA modification, and mRNA processing (Fig. 4e). Enriched GO terms for active genes with a higher Group 2 remodeller signal include those associated with mitochondrial processes such as ATP synthesis and respiratory chain activity (Fig. 4e). Together, this demonstrates that the Group 1 and Group 2 remodellers maintain and associate with the regulation of different cellular pathways.

### Group 1 and Group 2 remodellers are defined by chromatin architectural features

**Fig. 5:**
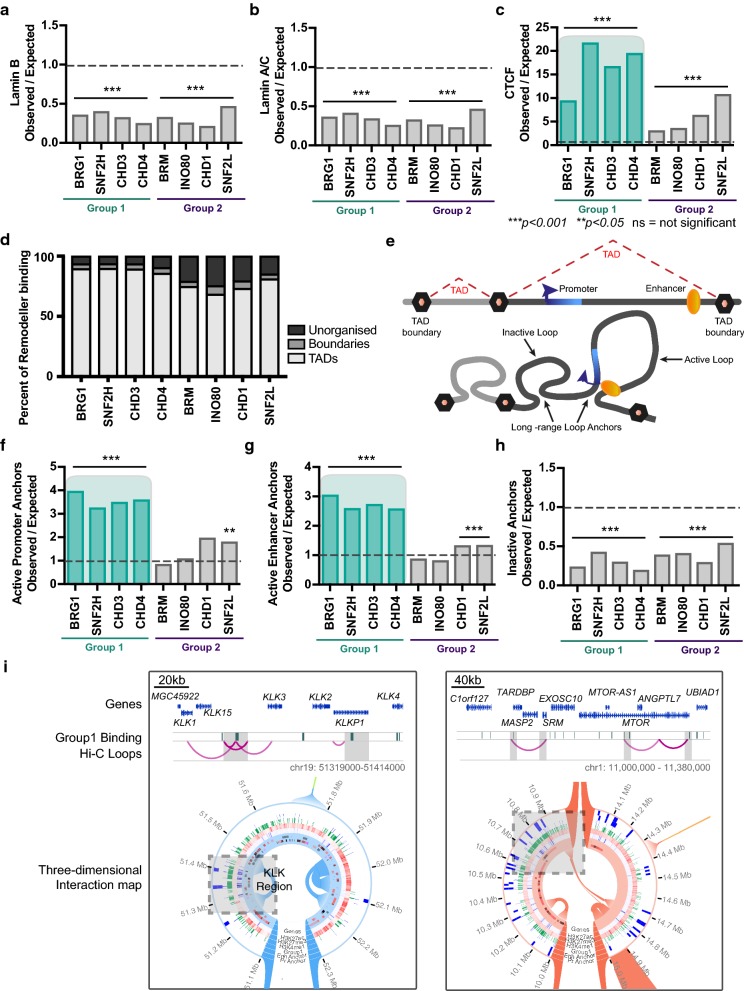
Chromatin remodeller binding at chromatin architectural features. **a**–**c** Enrichment of chromatin remodeller binding sites at the binding sites of key chromatin architectural proteins as defined by their ChIP-seq-binding profiles. Significant enrichment is defined as a score above one and significantly depleted as below one with Benjamini–Hochberg adj p-value, ***p < 0.001 or **p <0.05. **d** Percentage of remodeller binding sites in TADs, TAD boundaries and unorganised chromatin. **e** Schematic diagram depicting TADs in linear and 3D space with an inactive loop condensing chromatin and an active loop bringing an enhancer (orange oval) and promoter (blue flag) within close proximity. **f**–**h** Enrichment of chromatin remodeller binding at anchor points of chromatin loops defined by Hi-C and histone modification data as containing an active promoter or enhancer or neither. Significant enrichment is defined as a score above one and significantly depleted as below one with Benjamini–Hochberg adj p-value, ***p <0.001 or **p < 0.05. **i** The KLK region on chromosome 19 and a regions on chromosome 1 containing active loops (pink arcs) with Group 1 remodeller binding sites at the long-range loop anchors shaded in grey, with complementary three-dimensional view from Rondo (Rondo.ws)

The above results determined the positioning of remodellers at regulatory features in the context of histone post-translational modifications and DNA methylation in linear genomic space. However, chromatin is organised into a higher order by the formation of topologically associated domains (TADs) [53] that function to bring distal regulatory regions together for coordinated gene expression. Architectural proteins Lamin A/C, Lamin B and CCCTC-binding factor (CTCF) contribute to the formation of this three-dimensional chromatin structure. Lamin B and Lamin A/C configure the chromatin into Lamin-associated domains (LADs), with low accessibility [54]. LADs occur at the nuclear periphery, are heavily marked with H3K9me3 and are typically associated with gene repression. Therefore, we hypothesised that Group 2 remodeller binding will co-localise with LADs. Instead, using ChIP-seq data of Lamin B and Lamin A/C binding sites [55], we found all of the chromatin remodellers were significantly *depleted* (*p* < 0.001), suggesting that the tightly compacted chromatin at the nuclear periphery does not require remodeller binding to maintain its repressive state (Fig. 5a, b). Similarly, CTCF has diverse roles in regulating chromatin and the epigenome, and it is well established that nucleosomes directly flanking CTCF-binding sites are highly ordered, displaying a strong phasing pattern [48, 49, 56]. Previous research has shown that the ISWI remodeller protein SNF2H, but not the alternative ISWI ATPase SNF2L, contributes to this highly ordered array of nucleosomes around sites of CTCF occupancy when bound to chromatin [57]. Using CTCF ChIP-seq data, we found that similar to the ‘actively marked’ DNA regulatory elements, all remodelers were enriched at CTCF-binding sites, but Group 1 remodellers were more highly enriched compared to Group 2 (Student’s unpaired T-test, *p *=0.018), with SNF2H displaying the highest level of enrichment (Fig. 5c).

We next examined the distribution of chromatin remodellers within the context of higher-order three-dimensional chromatin structure itself using Hi-C data from LNCaP cells [29]. We divided chromatin into TADs (85.4% of the genome), TAD boundaries (2.6% of the genome) and unorganised chromatin (12.0% of the genome; Additional file 1: Figure S8A) and examined the distribution of Group 1 and Group 2 remodellers within each category. Remarkably, we found that 90% to 94% of Group 1 remodellers and 75% to 85% of Group 2 remodeller binding sites were located within TADs or at TAD boundaries, indicating that chromatin remodellers preferentially localised to sites of highly organised chromatin (Fig. 5d). We performed the GAT analysis and found a small but positive and significant enrichment of all eight of the remodellers at both TADs and TAD boundaries (Additional file 1: Figure S8B–C), which was expected as the majority of the genome is within these organised chromatin structures.

Within TADs, chromatin forms DNA loops to facilitate interactions such as those between enhancers and promoters (Fig. 5e), and these loops are in part affected by the positioning of nucleosomes [58, 59]. Our previous work has shown cancer-specific anchor points of long-range chromatin ‘loops’ are enriched for enhancers and promoters and contain a remodelled epigenetic signature, where ‘active’ marks H3K4me1, H3K4me3 and H3K27ac are increased [29]. Whether chromatin remodellers are enriched at the anchor points of these chromatin loops remains unknown. Our linear data suggest ‘active’ chromatin ‘loops’ that bring together promoters and enhancers would be more enriched for Group 1 remodellers compared to Group 2. To test this, we separated the anchor points of the long-range chromatin loops into those that contained at least one active promoter or active enhancer using the chromHMM chromatin state data (Type A anchors, ~ 20% of all anchors) and a second group that did not contain either of these regulatory elements (Type B anchors, ~ 80% of all anchors). We found that at Type A loop anchors, the remodellers continue to stratify into Group 1 and Group 2, with Group 1 chromatin remodellers significantly (*p *<0.001) more enriched than Group 2 (Fig. 5f, g). Interestingly, Type B loop anchors that were devoid of an active promoter or enhancer were significantly depleted (*p* < 0.001) of all chromatin remodellers (Fig. 5h), suggesting that these anchors do not require ongoing chromatin remodelling activity. Examples of chromatin loops with Group 1 remodeller binding and active regulatory elements are found at the *KLK* locus on chromosome 19, and a gene dense region on chromosome 1 (Fig. 5i). Together, this demonstrates a role for all Group 1 remodellers in chromatin three-dimensional architecture.

## Discussion

**Fig. 6:**
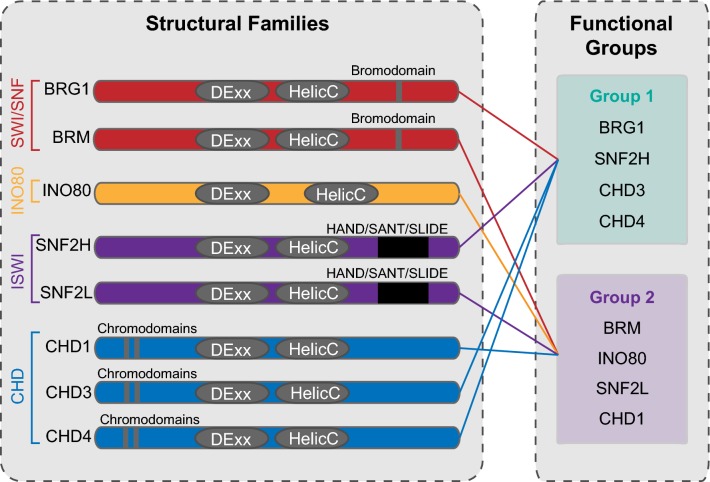
Schematic of chromatin remodeller families. Chromatin remodellers are divided firstly by their known structural families, which are then arranged by the groups they stratify into based on their binding enrichment from the epigenome

Chromatin stores important epigenetic information that is inherited across cell divisions. ‘Reader’ proteins, such as ATP-dependent chromatin remodelling complexes, are required to interpret and modify chromatin when required. While several studies demonstrate that chromatin remodeller complexes have strong ties with regulatory chromatin [15, 23, 25, 27, 60, 61], little is known about how these complexes function as a group and interact with the different layers of the epigenome. We sought to address this interrelationship through the use of publically available data by examining the role of eight different chromatin remodeller proteins in LNCaP prostate cancer cells and integrating their binding profiles with multiple epigenomic data sets. This is the first extensive analysis of multiple chromatin remodellers together, with several layers of the epigenome at the same time in human cells. We found that there is substantial overlap in the binding sites of the remodellers at regulatory regions of the genome. Specifically, we found that the eight remodellers can be stratified into two distinct groups: Group 1 that was more highly enriched at ‘actively marked’ chromatin regions and Group 2 at ‘repressively marked’ chromatin regions (Fig. 6).

Our data revealed that the grouping of chromatin remodellers is remarkably consistent across all of the ‘actively marked’ and ‘repressively marked’ epigenetic features we examined but intriguingly, independent of the core modification of DNA methylation. Segregation into these two groups persists at both defined DNA regulatory elements and chromHMM chromatin states, which are a composite of histone modification marks. While Group 1 remodellers have a significantly higher enrichment than Group 2 at ‘actively marked’ regions, it is still noteworthy that all remodellers have some level of enrichment at these regions, which highlights the dynamic and complex nature of active chromatin. Previous research also demonstrates a role of Group 2 remodellers at active chromatin in embryonic mouse cells such as INO80 in maintaining open chromatin in pluripotency genes [62] and CHD1 and its role in RNA polII stalling in active gene expression [63]. We also note that we find some divergent results for the remodeler CHD1. A previous study in mouse embryonic stem cells found CHD1 to only be enriched at active promoters [27]. In our study, CHD1 had an equivalent level of enrichment at active promoters as the Group 1 remodellers, while still being enriched at ‘repressively marked’ chromatin together with the other Group 2 remodellers. These differences may be due to the different cell types, embryonic cells versus somatic cells, or may reflect differences between normal versus cancer cells. It will be interesting to interrogate these differences in future studies.

It was surprising that upon investigation of the direct overlap within Group 1 and Group 2 remodeller binding sites, we found less than 6% were in common for the entire group. This suggests that, while remodellers within the same group have high statistical enrichment at the same class of regulatory elements, they do not always bind to the exact same genomic region. For example, Group 1 remodellers demonstrate a preference for ‘actively marked’ promoters as a collective group, but they often localise to their own subset of all active promoters, showing that each remodeller potentially has a distinct and unique role. Additional data will be needed to further resolve the types of complexes each of the core remodeller proteins is capable of forming. Expansion of the existing ChIP-seq data to include various accessory subunits will help refine this analysis, in combination with fine-tuning existing definitions of DNA regulatory elements and chromHMM states. Broadly defined, the further subdivision of DNA regulatory elements and states will enable subtyping and will provide additional details to determine under which conditions, states and combinations these remodeller proteins act in a coordinated or antagonistic manner.

For the purposes of this study, we did not include all known histone modifications in our analysis, including methylation of H4K20 and H4K16ac [64–66]. Thus, the proportion of remodeller binding found at ‘unmarked’ regions may contain histone modifications not present in our analysis. However, the key marks for defining DNA regulatory elements and genes were included and therefore provide a comprehensive view of key gene regulatory chromatin features, and moreover, less than 20% of binding sites fell within the ‘unmarked’ regions. As more histone modification data become available, such as histone variants and histone modifications with a structural role, it will be interesting to determine whether these also stratify the remodellers in a similar fashion.

Interestingly, we found that remodellers consistently segregated into Group 1 and Group 2 at architectural chromatin features, such as chromatin loop anchors and CTCF sites, highlighting that chromatin remodellers are also associated with higher-order chromatin architecture. Previous to this study, BRG1 was the sole remodeller that had a demonstrated role in chromatin architecture and a well-established role in maintaining enhancer–promoter interactions [11, 60, 67]. Additionally, in MCF10A cells, BRG1 increases the stability of TAD boundaries to strengthen enhancer–promoter chromatin loops and maintain the established patterns of gene expression. Subsequent loss of BRG1 binding weakens these interactions, concomitant with a down-regulation of gene expression [68]. Our results infer that in fact, all Group 1 remodellers—BRG1, SNF2H, CHD3 and CHD4—could have individual or a combinatorial role at chromatin loop anchors that contain active promoters or enhancers. When we examined the remodellers at LADs, however, we found them to be depleted of all remodellers. We also investigated the association with the architectural protein CTCF and found that Group 1 remodellers are more enriched at CTCF-binding sites compared to Group 2. Together, our data suggest Group 1 remodellers have a prominent role at ‘active’ chromatin loops, whereas ‘inactive’ chromatin loops do not require remodeller binding to maintain their repressed state.

Our finding that the distribution of chromatin remodellers across CpG islands follows three distinct patterns (BRG1, SNF2H and CHD4 at borders; CHD3 and CHD1 in the centre and SNF2L, INO80 and BRM deplete from the centre of the island) suggests that there are different mechanisms at play for maintaining nucleosome positions at these regions. It was surprising that neither CHD3 nor CHD4 showed any level of enrichment at methylated CpG islands. These remodellers form a key part of the NuRD complex that also contains MBD2, which prefers hypermethylated promoters [42]. It is possible that once the DNA of any given genomic region becomes methylated and the chromatin is compacted, it may no longer require the remodelling complex to stay bound to the chromatin.

Previous studies that have examined two to three remodeller proteins show consistency with our findings. The overlap of BRG1 and CHD4 has previously been reported in various cell types, where they were reported to have opposing control over regulatory chromatin [23, 26, 69, 70]. For example in mice, BRG1, CHD4 and SNF2H have extensive overlap in their binding patterns and it has been implied that the sequential order of their binding is important for their correct function [23]. In our data, we found that these remodeller proteins were also enriched at the same genomic features, suggesting they may also have opposing functions in LNCaP cells. Additionally, we found overlapping enrichment patterns for CHD1 and ISWI remodeller SNF2L, which in yeast have been reported to have both competing and coordinated functions. CHD1 and SNF2L are responsible for maintaining the phasing of nucleosomes at promoters, but compete for different nucleosome spatial arrangements, impacting the kinetics of gene activation [15, 24, 25, 71]. CHD1 and SNF2L also work together at gene bodies where they are thought to maintain chromatin integrity during transcription elongation by preventing histone exchange during nucleosome turnover [72–74]. Moreover, there has been report of overlapping activity between the ATPase subunits of the NuRD remodelling complex, CHD3 and CHD4 [20], which also occurred in our data.

Functional studies in human cancers have demonstrated the integrated relationships of chromatin remodellers, through the identification of synthetic–lethal relationships. Synthetic–lethal relationships occur where the cancer develops a loss of function for one protein that creates a dependency on another protein. Synthetic–lethal relationships exist for ATPases, BRG1 and BRM, in triple negative breast cancer, for accessory subunits of the SWI/SNF complex, ARID1A and ARID1B, in colorectal cancer and CHD1 with transcriptional regulator PTEN in prostate cancer [75–77]. These instances of the relationship between remodellers and their function highlight the importance of studying multiple remodellers together to provide a greater understanding of the overall mechanism of chromatin remodelling and how a chromatin state is established. It is unclear from our data how much of the remodeller binding overlap provides cooperative or antagonist function; moreover, our study does not determine the extent of competition between the remodellers for nucleosome binding. Mechanistic studies are now required, such as using gene editing approaches and in different cell model systems, to further dissect the importance and or redundancy of individual remodellers and their potential functional role in regulating chromatin, and broaden the applicability of findings to other cell types.

## Conclusions

In summary, our results reveal previously unknown relationships between the remodellers, and with both the two-dimensional and three-dimensional epigenome. We propose that chromatin remodellers should be examined in the context of the different classes of remodellers we identified as Group 1 or Group 2, and not solely with consideration to existing structural families or in isolation (Fig. 6). These observations may inform decisions for future work that studies chromatin remodeller function and provides a more complete picture of chromatin remodelling action.

## Methods

### ChIP-seq assay and data

LNCaP chromatin remodeller ChIP-seq data are from Ye et al. [28]: GEO accession GSE72690. Histone modification LNCaP ChIP-seq data (H3K4me3, H3K4me1, H3K27ac and H3K27me3) are from Taberlay et al. [29]: GEO accession GSE73785, and Bert et al. [34]: GSE38685. CTCF ChIP-seq data is from Bert et al. [34]: GSE38685. H3K9me3, H3K36me3, Lamin B and Lamin A/C ChIP-seq data are from Du et al. [55], GSE98732. p300 ChIP-seq data is from Wang et al. [78]: GSE27824. RNA polII ChIP-seq data is from Tan et al. [79]: GSE28264. All ChIP-seq data sets were processed as previously described in Bert et al. [34], Taberlay et al. [29], Du et al. [55] and Lund et al. [80]. Histone modification and chromatin remodeller peaks were called using MACS2 [81] and Lamin domains called with the enriched domain detector (EDD) [80]. Two ChIP-seq input data sets were provided by Ye et al. [28], which were merged for calling remodeller peaks. Then, MACS2 [81] was used to call peaks on each individual input data set; any peaks overlapping with the chromatin remodellers were removed from the remodeller data sets.

### Hi-C data

LNCaP Hi-C data is from Taberlay et al. [29]; GSE73785. Hi-C data were processed through the NGSANE framework v0.5.2 [82] as previously described in Taberlay et al. [29]. TADs were identified with the ‘domain-caller’ pipeline [53] as described in Taberlay et al. [29]. TADs and TAD boundaries were assessed at 40 kb resolution. The percentage of the genome covered by TADs and TAD boundaries was calculated by accumulating the number of base pairs within TADs or boundaries, divided by 3.095 × 10^6^ and then multiplied by 100. Chromatin loops were called from contact count matrices for 10 kb resolution using a custom adaptation of Fit-Hi-C (contained in NGSANE; Buske et al. [82]) supplying iteratively corrected bias offsets calculated through HiCorrector v1.1 [83]. Chromatin loops were visualised in the WashU Epigenomics Browser [84] and Rondo (rondo.ws).

### RNA-seq data

LNCaP RNA-seq data are from Taberlay et al. [29]: GSE73785, and processed clinical prostate tumour RNA-seq was downloaded from TCGA (cancergenome.nih.gov). LNCaP RNA-seq data (*n* = 3) were processed as described in Taberlay et al. [29]. To determine chromatin remodeller gene expression, reads mapped to hg19/GRCh37 where counted into genes using featureCounts [85] program and GENCODE v19 used as a reference transcriptome to determine the transcript per million read (TPM) value and biological triplicates were averaged. Processed RNA-seq data (*n* = 486 tumours) from the TCGA prostate adenocarcinoma cohort were averaged to determine chromatin remodeller expression in clinical prostate cancer samples. The log mean values for each remodeller were plotted as cancer versus normal between the cell lines (logTPM) and the normal and tumour TCGA data sets (logRKPM), along with the linear regression line of best fit. Pearson’s correlation coefficients were calculated in R.

### Chromatin accessibility data

DNaseI data is from Thurman et al. [47], and processed data were downloaded from the ENCODE data portal (encodeproject.org/). DNaseI sites of accessibility from two biological replicates overlapped and the intersection from both replicates were used for downstream analysis (see Remodeller enrichment analysis). NOMe-seq data are from Valdes-Mora et al. [86]: GSE76334. NOMe-seq data were processed as previously described in Valdes-Mora et al. [86]. NOMe GpC methylation levels within remodeller binding sites were defined by first computing the methylation ratio of all GCH sites with greater than 5× coverage and then calculating the mean methylation score within each remodeller binding site. Methylation density of remodeller binding sites was plotted in R. Visualisation of nucleosome occupancy at remodeller binding sites was created using ‘methylationPlotRegions’ from the *aaRon* package in R, ± 3000 bp from the centre of the remodeller binding site.

### DNA methylation data

WGBS sequencing data is from Pidsley et al. [46]: GSE86833. WGBS libraries were processed as previously described Pidsley et al. [46]. CpG islands and Ensembl gene coordinates were downloaded from the UCSC genome browser [87]. Promoter CpG islands were defined as the intersection between CpG islands and the 5′ ends of Ensembl genes. The promoter CpG islands were split into 40 equally sized bins and the average methylation score calculated from the WGBS data using ‘ScoreMatrixList’ from the *genomation* package in R. Methylated CpG islands were defined as having an average methylation score above 50%. DNA methylation levels within remodeller binding sites were defined by first computing the methylation ratio of all cytosines with greater than 5× coverage and then calculating the mean methylation score within each remodeller binding site. CpG density of methylated and unmethylated CpG islands was calculated in R. Violin and boxplots plots were created in *ggplot2* in R. Remodeller binding sites were overlapped with the methylated and unmethylated islands using the *GRanges* package in R.

### ChromHMM segmentation based on Roadmap epigenomics

The Roadmap Epigenomics chromHMM model was based on five core histone modifications (H3K4me3, H3K4me1, H3K36me3, H3K27me3, H3K9me3) and trained on 60 epigenomes [88] to categorise the genome into 15 chromatin states. There were seven chromatin states associated with ‘active’ chromatin; active promoter, flanking active promoter, transcription at 5′ and 3′ ends of a gene, strong transcription, weak transcription, active intragenic enhancers, active intergenic enhancers and poised enhancers. Three states were associated with bivalent chromatin: bivalent promoter, flanking bivalent promoter and bivalent enhancers. There were four states associated with ‘repressive’ chromatin: zinc finger genes and repeats, heterochromatin, strong polycomb and weak polycomb. The 15th state was ‘unmarked’ chromatin that did not contain any of the histone modifications in the core data set. This model was applied to histone modification ChIP-seq data from LNCaP cells using the chromHMM program (v1.10) [35], and the chromatin states were collapsed into ‘active’, ‘repressed’, ‘bivalent’ and ‘unmarked’.

### Chromatin remodeller enrichment and correlation analyses

To determine whether chromatin remodeller binding sites were enriched at the site of a specific chromatin factor or chromatin regulatory element (histone modifications, CTCF sites, LADs, DNaseI sites, chromatin loop anchors, TADs, TAD boundaries, methylated and unmethylated CpG islands and chromHMM states), we used the genome association tester (GAT; v1.0) [36]. The observed-over-expected fold change and statistical significance were calculated with 10,000 iterations and determine significant if the *p* value was less than 0.05 or 0.001. The difference between the means of the observed/expected enrichment scores from Group 1 and Group 2 remodellers was compared using the unpaired Student’s T-test in R.

We defined the percentage of overlap between each of the remodellers, and the remodellers with TADs and TAD boundaries by intersecting the peaks identified from the ChIP-seq and Hi-C data. Histone modification average signal plots over chromatin remodeller peaks and the heatmaps of putative active enhancers and chromatin remodeller signal over CpG islands and promoters were created with SeqPlots [89] and deepTools2 [90]. Histone marks were plotted ± 2 kb from the centre of the remodeller binding sites. At CpG islands remodeller ChIP-seq signal for both average plots and heatmaps were plotted ± 2 kb from the centre of the island. Each row of the heatmap is an individual CpG island and displays the remodeller ChIP-seq signal, sorted by the average signal across all remodellers in decreasing order. Pearson’s correlation matrixes of remodellers at promoters and DNaseI sites were calculated in R.

### Gene ontology enrichment

Gene promoters were defined as 2 kb surrounding the TSS of expressed genes. Read counts of the chromatin remodeller ChIP-seq data within the promoters regions of expressed genes were calculated and all Group 1 remodellers merged and separately Group 2 remodellers merged. Subtracting the total read counts of Group 2 remodellers from Group 1 was used to define which promoters had a higher signal for Group 1 and which had a higher signal for Group 2. The promoters assigned to each group were analysed for enrichment of gene ontology terms using GREAT [52], using the whole genome as background and assigned to the single nearest gene. GO terms reported are the top 10 most significant from the Molecular Function, Biological Process or Cellular Component gene set, with at least 25 observed genes in the data set.

## Additional file

**Additional file 1: Figure S1.** A) Gene expression of each remodeller protein in LNCaP cells from RNA-seq (mean and SE; n = 3). B) Gene expression of remodeller proteins in TCGA data set of 486 prostate epithelial tumours (mean and SE; n = 486). C) Gene expression of each remodeller in PrEC cells from RNA-seq (mean and SE; n = 3), D) Gene expression of remodellers in TCGA data set of normal prostate epithelial tissue (mean and SE; n = 52). E) Scatter plot of logRPKM values for TCGA data versus logTPM values for LNCaP and PrEC RNA-seq data. The PrEC comparison for PrEC and normal TCGA is shown in green, and the LNCaP compared to TCGA tumours is shown in red. Lines are linear regression line of best fit, and Pearson’s correlation coefficient between the two normal data sets (*cor *=* 0.670013*) and the two cancer data sets (*cor *=* 0.8375552*, SNF2L is excluded as an outlier) is shown under the plot. **Figure S2.** A–H) Histograms of chromatin remodeller binding sites, binned at 150 bp widths. Vertical line indicates the point of 750 bp (~ 5 nucleosomes). **Figure S3.** A) Heat map of ChromHMM emission profile based on the learned model from the epigenome roadmap. B) Heat map of chromatin state enrichment generated from the chromHMM analysis, where the percentage of each state in the genome is represented in the first column and the remaining columns are the enrichment of each chromatin state over annotated genomic features. C) Heatmap of H3K27ac, H3K4me1, H3K4me3 and p300 ChIP-seq signal and DNaseI signal at putative active enhancers, ± 2.5 kb from the centre of each enhancer, sorted by H3K27ac and H3K4me1 signal. **Figure S4.** A) Heatmap H3K4me3, RNA polII, and chromatin remodeller ChIP-seq signal and DNaseI signal at refseq-annotated promoters. Signal is plotted ± 2 kb from the transcription start site (TSS) and sorted by H3K4me3 signal. B, D) Venn diagram of chromatin remodeller binding site overlaps for Group 1 and Group 2 remodellers, respectively. C) Percentage of all Group 1 remodeller binding sites that are unique to each Group 1 remodeller and contain multiple Group 1 remodellers. E) Percentage of all Group 2 remodeller binding sites that are unique to each Group 2 remodeller and contain multiple Group 2 remodellers. **Figure S5.** A–O) Enrichment of chromatin remodeller binding sites across the 15 state chromHMM model based on the Epigenome roadmap (see Methods). Significant enrichment is defined as a score above one and significantly depleted as below one with Benjamini–Hochberg adj p-value, ***p <* 0.001* or **p <* 0.05*. **Figure S6.** A–D) Genome-wide average distribution of ChIP-seq signals for histone modifications H3K4me3, H3K4me1, H3K27me3 and H3K9me3 ± 2 kb from the centre of chromatin remodeller binding sites. E–H) Pearson’s correlation score matrix of chromatin remodeller ChIP-seq signal at active, bivalent, facultative and constitutive promoters. Each matrix was ordered by hierarchical clustering. **Figure S7.** A–B) Violin plots of nucleotide frequency within remodeller binding sites for Group 1 and Group 2. C–D) Dinucleotide frequency within chromatin remodeller Group 1 and Group 2 binding sites. E) DNA methylation density within chromatin remodeller binding sites for each remodeller. F) CpG density within chromatin remodellers binding sites for each remodeller protein. G) CpG density of unmethylated CpG islands, methylated CpG islands and the whole genome. A significant difference in CpG density was detected between each of the groups (one-way ANOVA, ***p <* 0.001*). **Figure S8.** A) The genome divided into TADs (85.4%), TAD boundaries (2.6%) and unorganised chromatin (12.0%) using TADs and boundaries called to a 40 kb resolution from Hi-C data. B–C) GAT enrichment of chromatin remodellers at TADs and TAD boundaries, where significant enrichment is defined as a score above one and significantly depleted as below one with Benjamini–Hochberg adj p-value, ***p <* 0.001* or **p <* 0.05*.

## Abbreviations

BRG1: Brahma-related gene 1
BRM: Brahma
CHD: chromodomain helicase DNA binding
chromHMM: chromatin multivariate hidden Markov model
CTCF: CCCTC-binding factor
H3K27ac: histone 3 lysine 27 acetylation
H3K27me3: histone 3 lysine 27 trimethylation
H3K4me1: histone 3 lysine 4 monomethylation
H3K4me3: histone 3 lysine 4 trimethylation
H3K9me3: histone 3 lysine 9 trimethylation
Hi-C: high-throughput chromosome conformation capture
INO80: inositol requiring 80 complex
SNF2: sucrose non-fermenting 2
SWI/SNF: switch/sucrose non-fermenting
