# Supplementary material for: Integrated epigenomic analysis stratifies chromatin remodellers into distinct functional groups
Source: Epigenetics Chromatin. 2019 Feb 12;12:12. doi: 10.1186/s13072-019-0258-9 (PMC6371444; doi:10.1186/s13072-019-0258-9)

SUPPLEMENTARY FIGURE 1

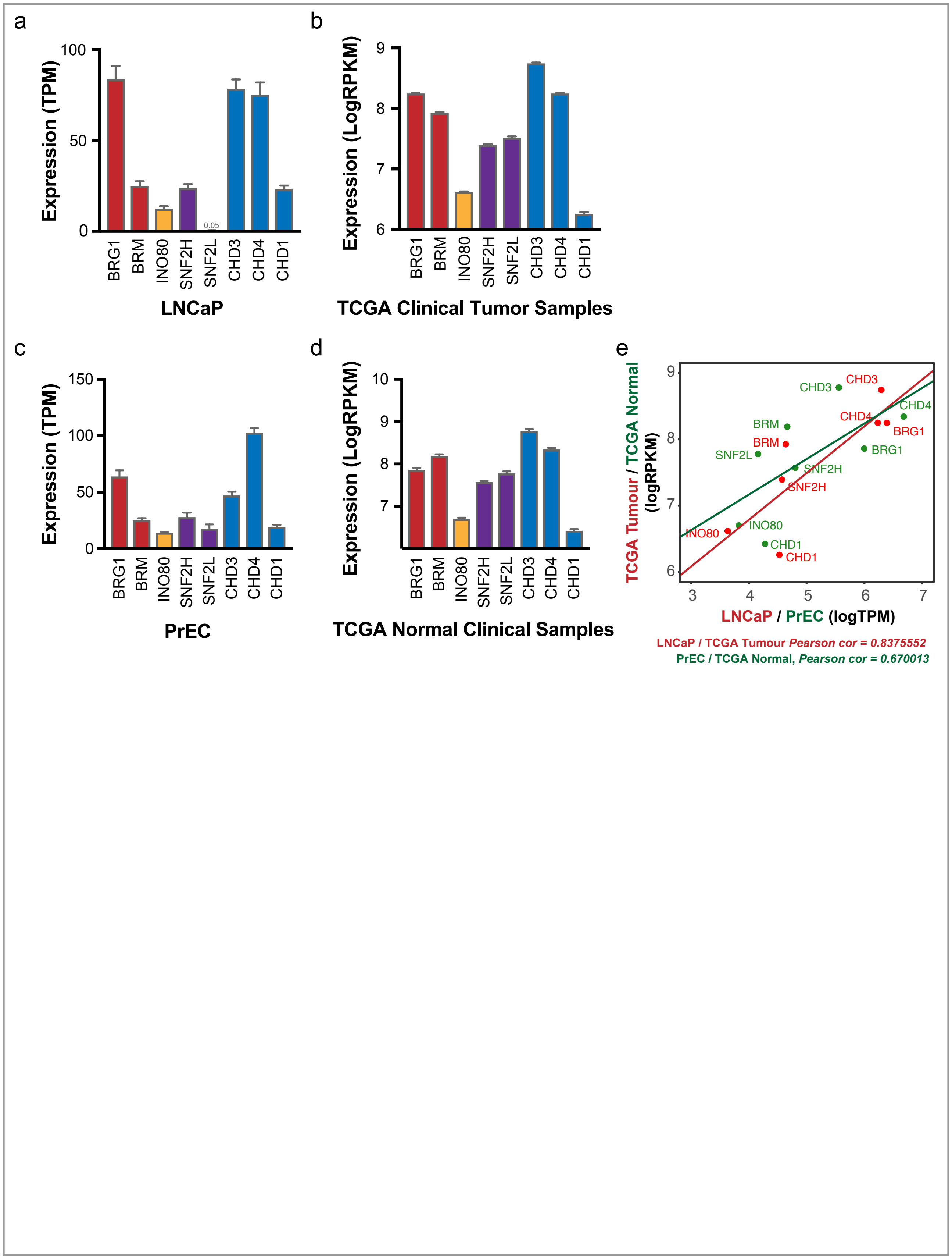

SUPPLEMENTARY FIGURE 2

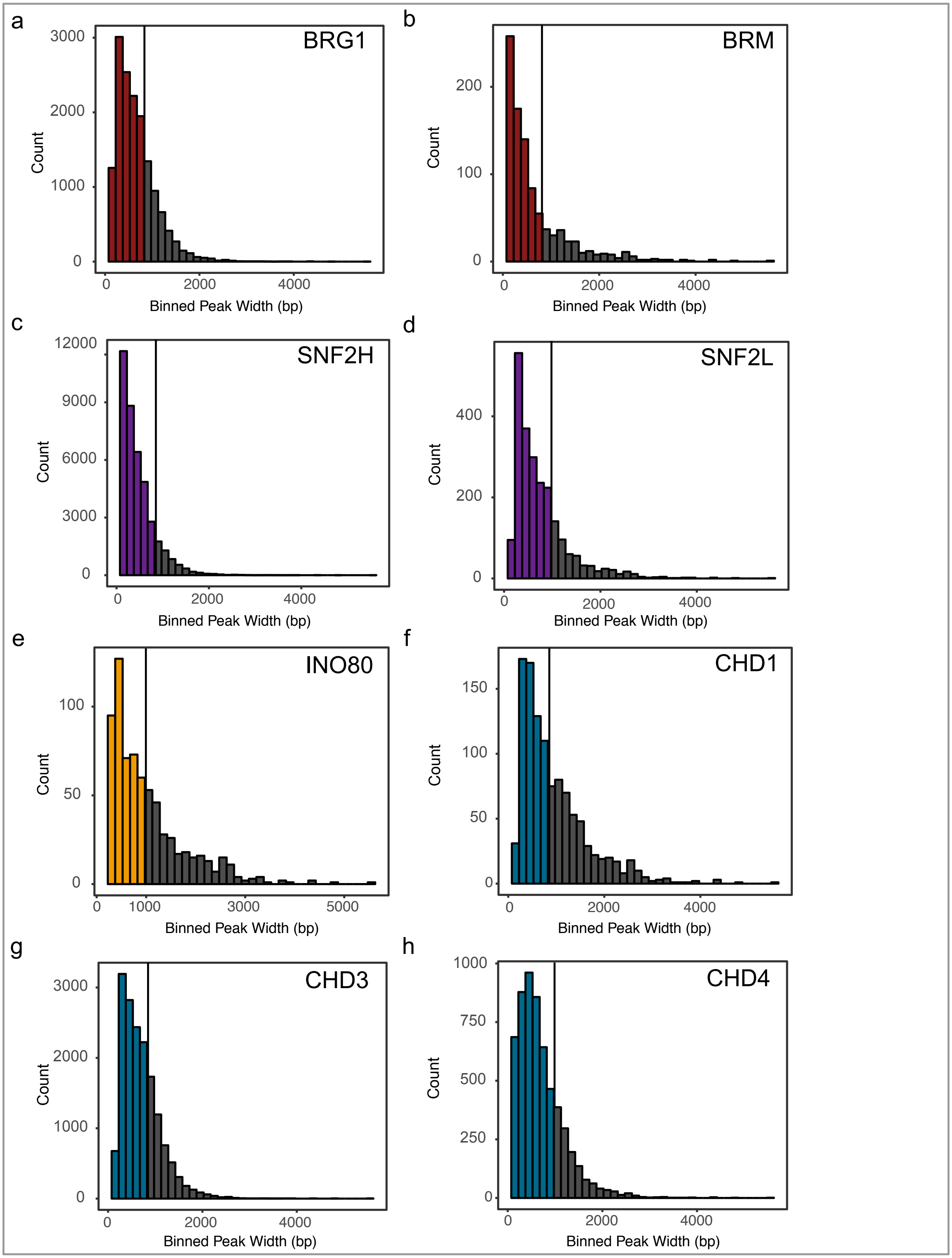

SUPPLEMENTARY FIGURE 3

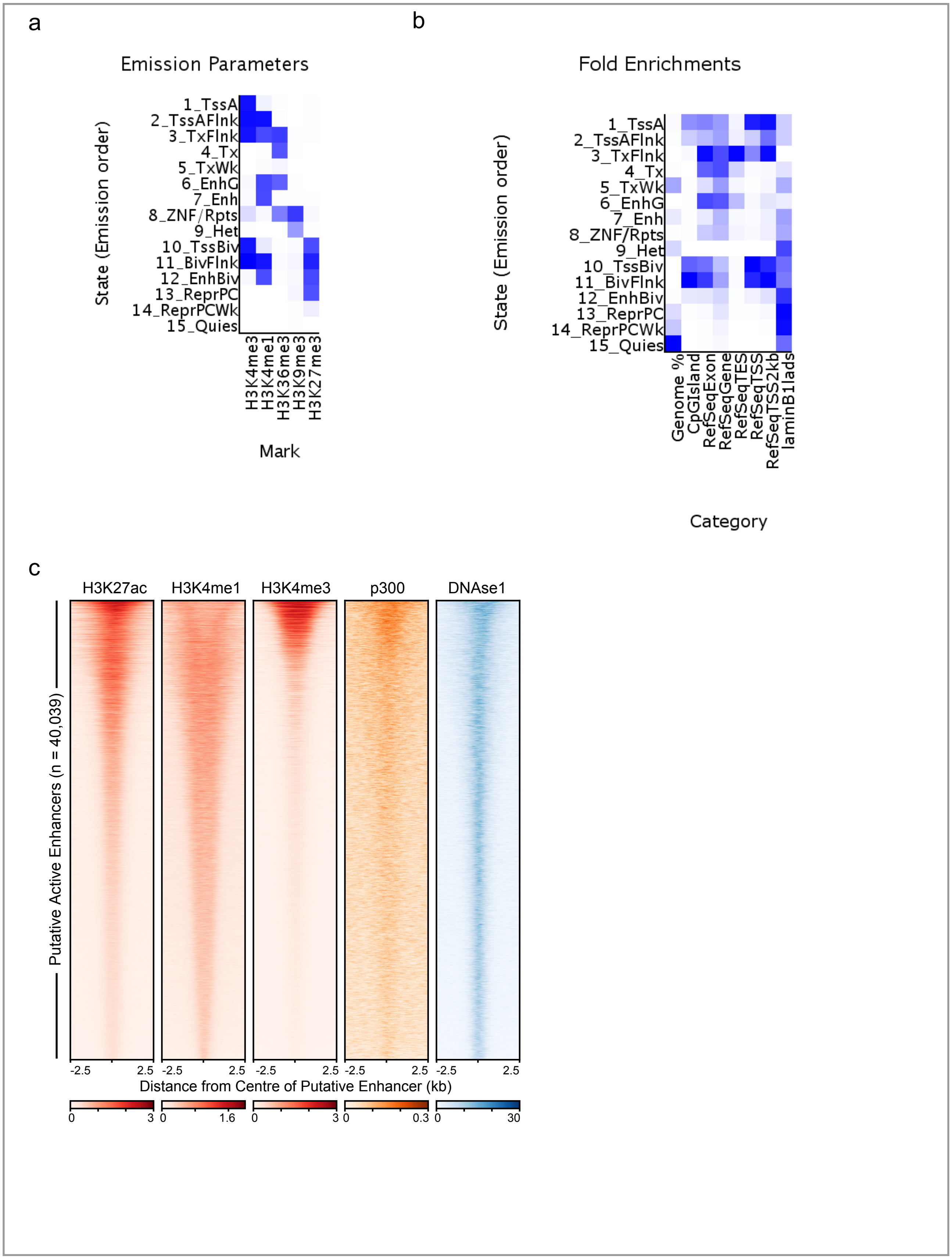

SUPPLEMENTARY FIGURE 4

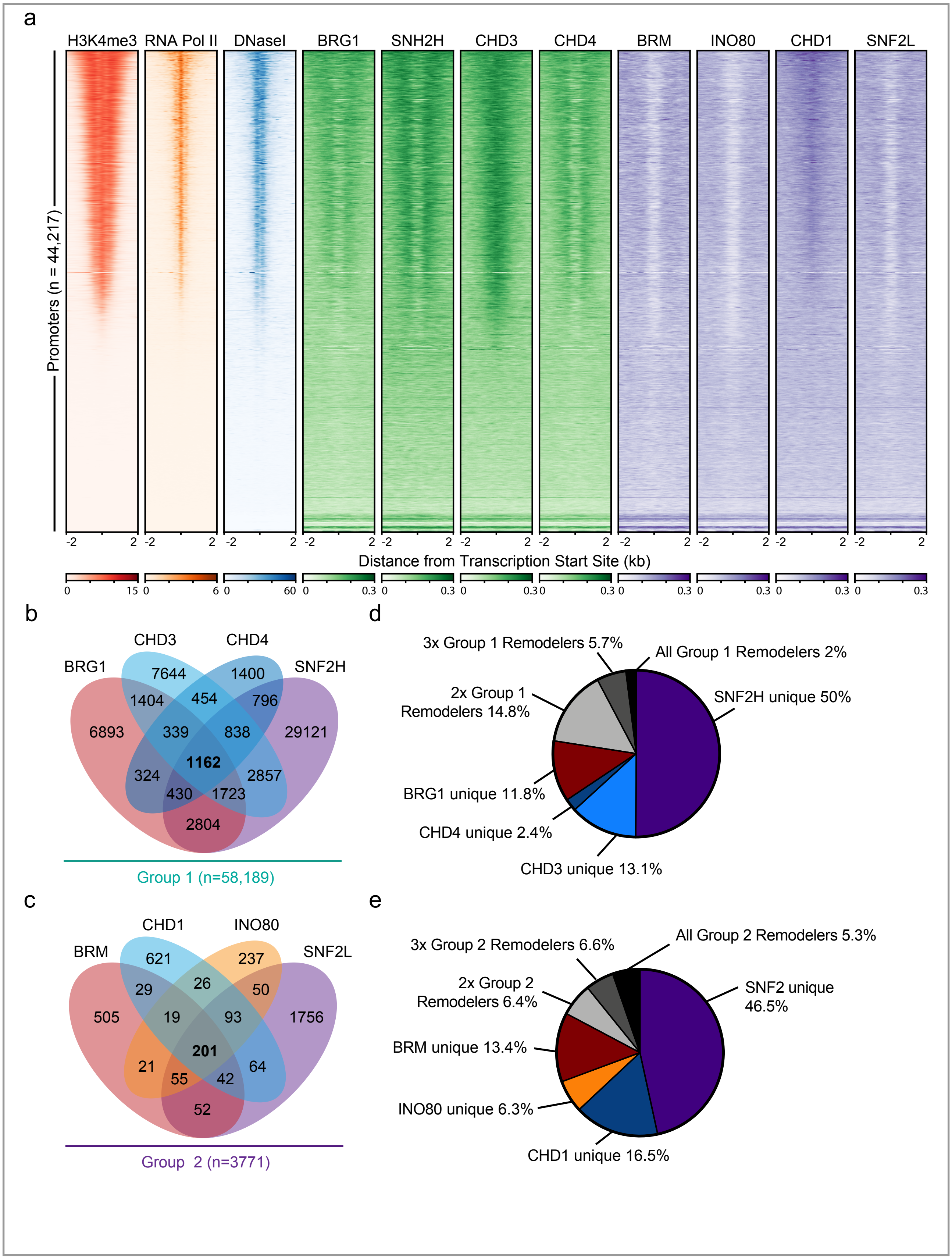

SUPPLEMENTARY FIGURE 5

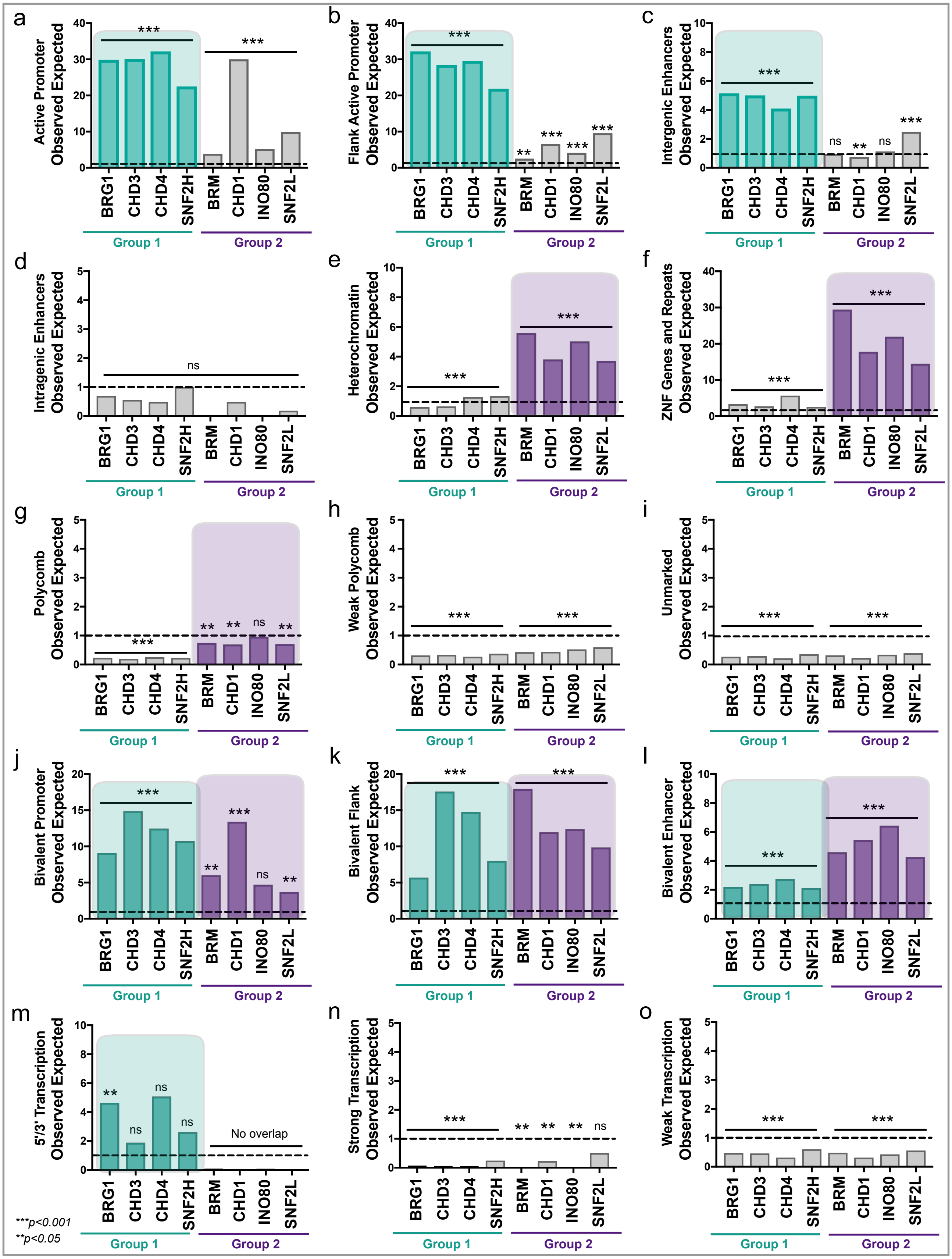

SUPPLEMENTARY FIGURE 6

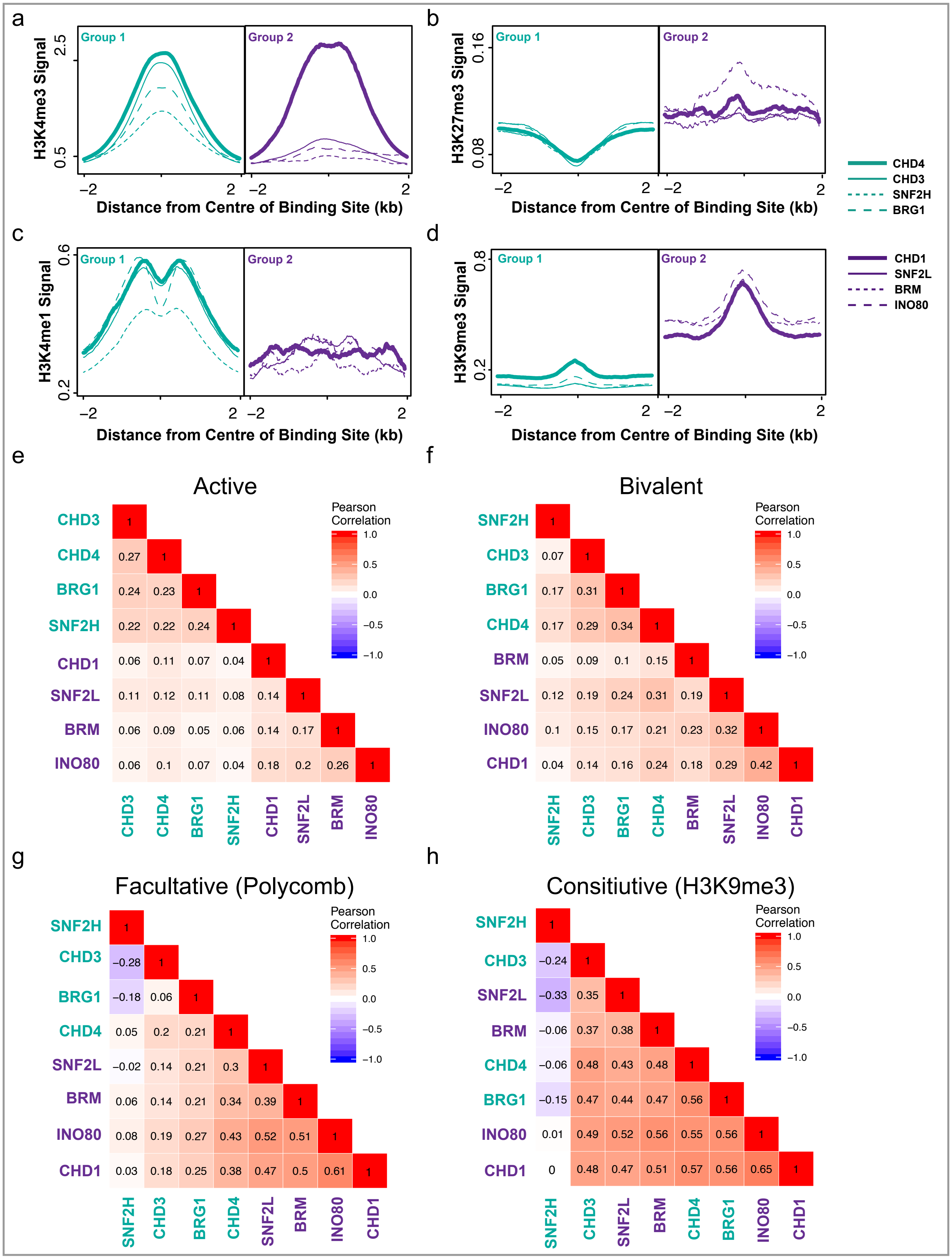

SUPPLEMENTARY FIGURE 7

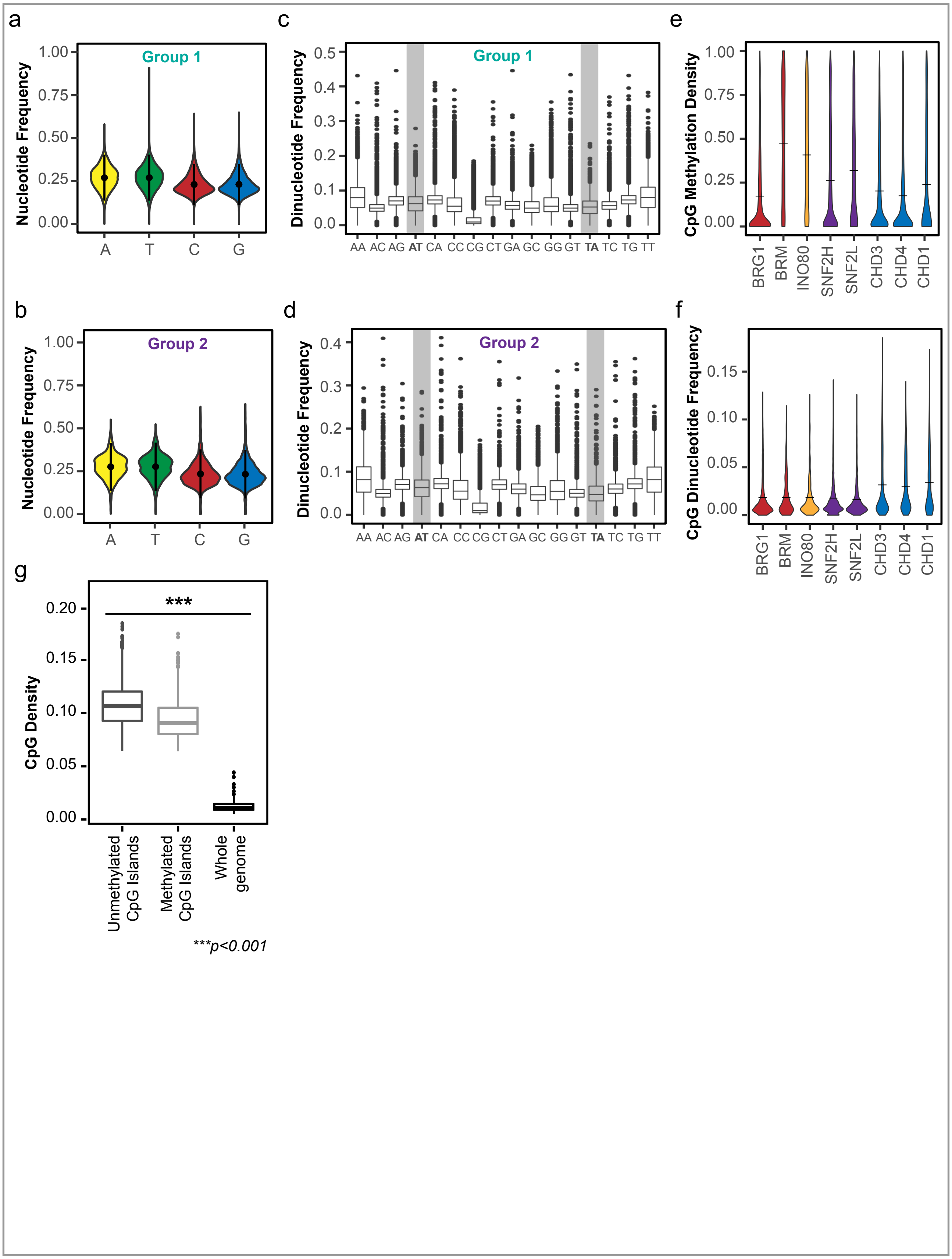

SUPPLEMENTARY FIGURE 8

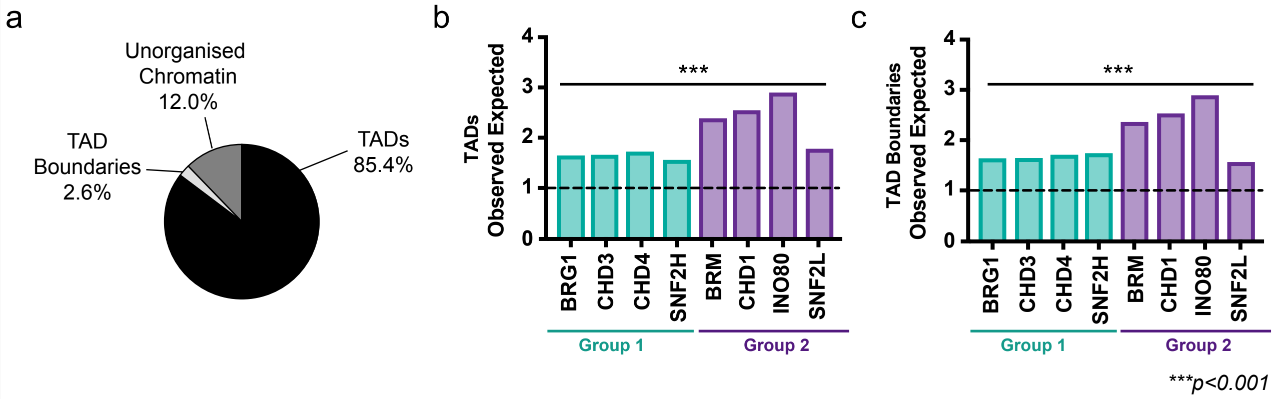

Supplement: Supplementary file 1 — Additional file 1: Figure S1. A) Gene expression of each remodeller protein in LNCaP cells from RNA-seq (mean and SE; n = 3). B) Gene expression of remodeller proteins in TCGA data set of 486 prostate epithelial tumours (mean and SE; n = 486). C) Gene expression of each remodeller in PrEC cells from RNA-seq (mean and SE; n = 3), D) Gene expression of remodellers in TCGA data set of normal prostate epithelial tissue (mean and SE; n = 52). E) Scatter plot of logRPKM values for TCGA data versus logTPM values for LNCaP and PrEC RNA-seq data. The PrEC comparison for PrEC and normal TCGA is shown in green, and the LNCaP compared to TCGA tumours is shown in red. Lines are linear regression line of best fit, and Pearson’s correlation coefficient between the two normal data sets (cor = 0.670013) and the two cancer data sets (cor = 0.8375552, SNF2L is excluded as an outlier) is shown under the plot. Figure S2. A–H) Histograms of chromatin remodeller binding sites, binned at 150 bp widths. Vertical line indicates the point of 750 bp (~ 5 nucleosomes). Figure S3. A) Heat map of ChromHMM emission profile based on the learned model from the epigenome roadmap. B) Heat map of chromatin state enrichment generated from the chromHMM analysis, where the percentage of each state in the genome is represented in the first column and the remaining columns are the enrichment of each chromatin state over annotated genomic features. C) Heatmap of H3K27ac, H3K4me1, H3K4me3 and p300 ChIP-seq signal and DNaseI signal at putative active enhancers, ± 2.5 kb from the centre of each enhancer, sorted by H3K27ac and H3K4me1 signal. Figure S4. A) Heatmap H3K4me3, RNA polII, and chromatin remodeller ChIP-seq signal and DNaseI signal at refseq-annotated promoters. Signal is plotted ± 2 kb from the transcription start site (TSS) and sorted by H3K4me3 signal. B, D) Venn diagram of chromatin remodeller binding site overlaps for Group 1 and Group 2 remodellers, respectively. C) Percentage of all Group 1 remo [file 13072_2019_258_MOESM1_ESM.pdf]
